# Bone marrow derived “fibrocytes” contribute to tumor proliferation and fibrosis in gastric cancer

**DOI:** 10.1007/s10120-014-0380-0

**Published:** 2014-05-04

**Authors:** Shiro Terai, Sachio Fushida, Tomoya Tsukada, Jun Kinoshita, Katsunobu Oyama, Koichi Okamoto, Isamu Makino, Hidehiro Tajima, Itasu Ninomiya, Takashi Fujimura, Shinichi Harada, Tetsuo Ohta

**Affiliations:** 1 Department of Gastroenterological Surgery, Kanazawa University Graduate School of Medical Science, 13-1 Takara-machi, Kanazawa, 920-8641 Japan; 2 Center for Biomedical Research and Education, Kanazawa University Graduate School of Medical Science, Kanazawa, Japan

**Keywords:** Fibrocytes, Bone marrow-derived cell, Cancer associated fibroblasts, Cancer stroma, Gastric cancer

## Abstract

**Background:**

Cancer-associated fibroblasts (CAFs) in the stroma are considered to play important roles for gastric cancer proliferation, invasion, and fibrosis, but the source of CAFs and their interaction with cancer cells in the microenvironment have not been fully determined. Here we elucidated the role of bone marrow-derived cells, fibrocytes, in development of gastric cancers, as represented by scirrhous gastric cancer.

**Materials and methods:**

In co-culturing MKN45 gastric cancer cells and purified fibrocytes from healthy volunteers, migration and endothelial mesenchymal transition associated gene expression were evaluated using western blot analysis. Also, mouse xenograft models of MKN45 with or without fibrocytes were conducted to investigate their tumorigenicity and immunohistological differences of tumors.

**Results:**

Co-culture of fibrocytes with MKN45 resulted in morphological changes from cobblestone-shape to spindle-shape and enhanced expression of α-SMA and collagen type I in fibrocytes, suggesting that co-culture with gastric cancer cells may have induced the differentiation of fibrocytes to myofibroblasts. Furthermore, enhanced expression of SDF-1 in MKN45 and CXCR4 in fibrocytes were also determined. Mouse xenograft models inoculated with MKN45 and fibrocytes revealed significantly larger tumors than those inoculated with MKN45 cells alone, and the stroma in co-inoculated tumors consisted of myofibroblasts and fibrosis. Mouse-derived cells expressing both CD45 and type I collagen were also observed in co-inoculated tumors.

**Conclusion:**

Fibrocytes derived from bone marrow may migrate into the microenvironment of gastric cancer by SDF-1/CXCR4 system, and enhance the tumor proliferation and fibrosis as CAFs.

## Introduction

Tumor tissues are not composed solely of cancer cells, but rather of a mixture of cell types and tissues. These mixtures of various cell types and extracellular matrix are known as the cancer stroma. Cells of the cancer stroma include fibroblasts, endothelial cells that constitute the vascular walls, immune cells such as lymphocytes and macrophages, and other bone marrow-derived cells. The interactions between cancer cells and the adjacent stroma form a complex tumor microenvironment [1–3].

Tumor cells release growth factors that promote tissue fibrosis, including transforming growth factor-β (TGF-β), platelet-derived growth factor, and fibroblast growth factor. These growth factors have been shown to activate fibroblasts in cancer stroma [4].

Fibroblasts and myofibroblasts in cancer stroma play critical roles in tumor proliferation and invasion [5, 6]. In general, fibroblast activation results in the over-expression of α-SMA, a biomarker for myofibroblast differentiation.

Fibroblasts in cancer stroma, called cancer-associated fibroblasts (CAFs), differ from conventional normal fibroblasts [7]. CAFs express several cytokine and chemokine receptors and can produce growth factors, cytokines, and chemokines. These molecules can induce the proliferation of cancer cells and their acquisition of invasive potential, as well as promote fibrosis. CAFs may originate from the differentiation of fibroblasts found in primary organs, or from bone marrow-derived cells that migrate to and differentiate in tumor tissue [8, 9].

Circulating bone-marrow-derived cells with fibroblast-like morphology, called fibrocytes, were found to migrate to sites of acute injury and contribute to the process of wound repair [10]. These fibrocytes accounted for 0.1–0.5 % of peripheral blood leukocytes in healthy individuals. Human fibrocytes derived from peripheral blood mononuclear cells can be cultured in plastic dishes, where they appear as adherent, spindle-shaped cells. Furthermore, fibrocytes express surface markers for hematopoietic stem cells (CD34) and a pan-leukocyte marker (CD45), while also producing extracellular matrix molecules including collagen types I and III and fibronectin [11, 12]. Fibrocytes have been reported to migrate into inflammatory tissues and differentiate into fibroblasts and myofibroblasts. Moreover, they are involved in wound-healing processes and in fibrotic diseases of the liver, lungs, and kidneys [13–17].

A high proportion of the tumor mass in patients with scirrhous gastric cancer and peritoneal dissemination is composed of cancer stroma. Although orthotopic fibroblasts may play important roles in the progression, growth, and invasion of gastric cancer [18], the sources of CAFs have not yet been fully determined. Bone marrow-derived cells have been reported to migrate to cancer stroma, where they differentiate into the myofibroblast phenotype [9], but the role of these bone-marrow-derived cells in cancer stroma is unclear. We have therefore assessed whether circulating fibrocytes can differentiate into CAFs and contribute to tumor proliferation and fibrosis.

## Materials and methods

### Cell lines and cell culture

Peripheral blood was collected from healthy volunteers into conical tubes containing 1–2 mL heparin, and mixed with an equal volume of saline. Peripheral blood mononuclear cells (PBMCs) were isolated using Lymphoprep Tubes (Axis-Shield, Dundee, Scotland) following the manufacturer’s protocol, and cultured in RPMI-1640 supplemented with 20 % heat-inactivated fetal bovine serum (FBS), penicillin, and streptomycin. After 2–4 days, the non-adherent cells were removed by gentle aspiration, and the media were replaced. After culture for an additional 10–12 days, the cells were harvested by incubation in ice-cold 0.05 % EDTA in phosphate-buffered saline (PBS). These crude fibrocyte preparations (about 70 % pure) were depleted of contaminating T cells, B cells, and monocytes by immunomagnetic negative selection using microbeads (MACS, Miltenyi Biotec, Bergisch Gladbach, Germany) coated with anti-CD2, anti-CD19, and anti-CD14 monoclonal antibodies, respectively [14]. To determine the purity of these fibrocyte preparations, the cells (5 × 10^5^/100 µL) were incubated in the dark for 30 min at 4 °C with a monoclonal mouse-anti human CD45 antibody conjugated to phycoerythrin (PE; BD Biosciences, San Diego, CA, USA) and a mouse anti-collagen type I antibody conjugated to fluorescein isothiocyanate (FITC; Rockland, Inc., Gilbertsville, PA, USA). The cells were washed twice with PBS and analyzed by flow cytometry using an Attune^®^ Acoustic Focusing Cytometer (Applied Biosystems; Life Technologies, Carlsbad, CA, USA) with Attune^®^ Cytometric Software.

The MKN45 human gastric cancer cell line, derived from a poorly differentiated adenocarcinoma, was purchased from the American Type Culture Collection (Manassas, VA, USA). These cells were seeded in 75-cm^2^ dishes (Becton–Dickinson, Franklin Rakes, NJ, USA) and cultured in 10 mL RPMI-1640 (Life Technologies Co., Carlsbad CA USA), supplemented with 10 % FBS (Nichirei Bioscience Tokyo, Japan), 100 IU/mL penicillin, and 100 mg/mL streptomycin (Life Technologies) at 37 °C in a humidified atmosphere of 5 % CO_2_ in air. After reaching confluence, the cells were harvested by trypsinization with 0.25 mg/mL trypsin/EDTA (Life Technologies) and suspended in culture medium before use.

### Differentiation of PBMCs and gastric cancer cells

MKN45 cells were seeded at a density of 1 × 10^5^ cells per well in 6-well plates and incubated for indirect co-culturing with the same number of fibrocytes using a 1 μm pore Boyden Chamber (Corning, Tewksbury, MA, USA) for 48 h, then cells were harvested for western blot analysis. PBMCs were seeded at a density of 2 × 10^5^ cells per well in 6-well plates containing medium supplemented with 20 % FBS. After 6 days, the cells were switched to serum-free culture medium for 24 h. As a control, cells were cultured for 48 h in medium containing 5 ng/mL recombinant human TGF-β1 (Sigma-Aldrich, St. Louis, MO, USA). Cell morphology and population were evaluated. Similarly, PBMCs were cultured alone or together with an equal number of MKN45 cells for 48 h, and the immunomagnetic cell sorting method was used to separate PBMCs from MKN45 cells. Following trypsinization, the cells were incubated with microbeads coated with anti-human CD326 antibody (Miltenyi Biotec, Bergisch Gladbach, Germany), because CD326 antigen is generally expressed on the surface of cancer cells, but not bone marrow-derived cells. Samples were assayed in triplicate and the results averaged.

### Western blotting analysis

Cells were lysed in RIPA buffer (50 mM, pH 8.0, Tris–HCl 150 mM NaCl, 0.5 w/v% sodium deoxycholate, 0.1 w/v% sodium dodecyl sulfate, and 1.0 w/v% NP-40 substitute) (Wako, Tokyo, Japan) containing 1 % protease inhibitor cocktail (Sigma-Aldrich). The protein concentration of each sample was measured using a BCA protein assay (Pierce Biotechnology, Rockford, IL, USA). Whole-cell lysates were prepared in denaturing SDS sample buffer, subjected to SDS-PAGE (ATTO Co. Ltd., Tokyo, Japan), and transferred to nitrocellulose membranes. These membranes were incubated with primary antibodies to CD34 (H-140, rabbit polyclonal IgG, diluted 1:1,000; Santa Cruz Biochemistry, Santa Cruz, CA, USA), COL1A1 (H-197, rabbit polyclonal IgG, diluted 1:1,000; Santa Cruz Biochemistry), αSMA (1A4, mouse monoclonal IgG, diluted 1:5,000; DakoCytomation, Copenhagen, Denmark), E-cadherin (H-108, rabbit polyclonal IgG, diluted 1:1,000; Santa Cruz Biochemistry), CXCR4 (C-20, goat polyclonal IgG, diluted 1:1,000, Santa Cruz Biochemistry), SDF-1 (C-19, goat polyclonal IgG, diluted 1:500, Santa Cruz Biochemistry), and β-actin (AC-15, mouse monoclonal IgG, diluted 1:10,000; Sigma-Aldrich). Antibody binding was visualized using ECL Western-Blotting detection kits (GE Healthcare, Waukesha, WI, USA) and the Light-Capture system (ATTO), and then quantified using the CS analyzer program (ATTO). All experiments were repeated three times.

### Mouse xenograft model

All animal experiments conformed to the guidelines of the Kanazawa University for the care and use of laboratory animals. BALB/c nu/nu female mice aged 4–5 weeks were obtained from Charles River Laboratories Inc. (Japan). Fibrocytes were stained with red fluorescent dye PKH26 (4 μM) using a cell linker kit (Sigma-Aldrich) according to the manufacturer’s instruction. Each of the eight control mice was inoculated 7 × 10^6^ of MKN45 cells in 100 μL of RPMI-1640 subcutaneously, whereas each of the eight experimental mice was inoculated 5 × 10^6^ of MKN45 cells plus 2 × 10^6^ of fibrocytes in 100 μL of RPMI-1640. Xenograft tumors were measured every day for 11 days. Tumor volume was estimated using the equation *v* = (*ab* ^2^)/2, in which *v* is volume, *a* is the length of the major axis, and *b* is the length of the minor axis. After 10 days, the mice were sacrificed, and the tumors were removed for immunohistochemical examination.

### Histological and immunohistochemical examination

Subcutaneous tumor specimens removed from xenografted mice were shock frozen in liquid nitrogen, cryosectioned, mounted onto glass slides, and air dried. The samples were analyzed by fluorescence microscopy using a standard filter setup for visualization of PKH26. Other samples were fixed in 10 % neutral buffered formalin, embedded in paraffin, and stained with hematoxylin and eosin or Mallory-Azan stain and observed under a light microscope. Other tissue samples were immunostained with antibody to α-SMA (1A4, mouse monoclonal IgG, diluted 1:100; DakoCytomation) overnight at 4 °C. The sections were treated with EnVision reagent (Dako Co., Japan) for visualization. In addition, sections were doubly immunostained with specific antibodies against CD45 and collagen type I to assess the numbers of mouse-derived cells by dual immunohistochemical techniques. The slides were immersed in methanol containing 0.3 % H_2_O_2_ for 30 min, blocked with 3.3 % normal goat serum in PBS and incubated with rat anti-mouse CD45 polyclonal antibodies (1:100; R&D Systems, Minneapolis, MN, USA) and rabbit anti-mouse collagen type I (1:100; Santa Cruz Biotechnology) for 2 h at room temperature. After washing in PBS, the sections were incubated with anti-rat IgG antibody conjugated with Alexa Fluor^®^ 488 and anti-rabbit IgG antibody conjugated with Alexa Fluor^®^ 546 (Molecular Probes/Life Technologies) (1:400) for 1 h at room temperature. Nuclei were counterstained with DAPI (diluted 1:1,000; Molecular Probes/Life Technologies) for 5 min and the slides viewed with an immunofluorescence microscope (BX50/BX-FLA; Olympus, Japan).

### Statistical analysis

Results are expressed as means ± standard deviations and compared using one-way analysis of variance or two-sided Student’s *t* tests. All statistical analyses were performed using the computer software package SPSS 10.0 (SPSS Inc., USA), with *p* < 0.05 indicating a statistically significant difference.

## Results

### TGF-β induced differentiation of PBMCs to fibrocytes and myofibroblasts

**Fig. 1:**
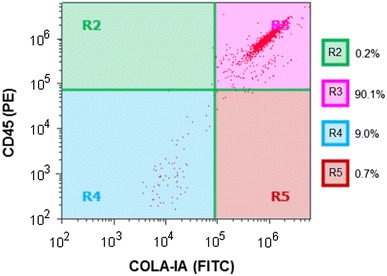
Preparation of fibrocytes. Crude fibrocyte preparations derived from PBMCs were depleted of contaminating T cells, B cells, and monocytes by immunomagnetic negative selection. The cells were stained with monoclonal mouse-anti human CD45 conjugated to phycoerythrin (PE) and monoclonal anti-human COLA-IA conjugated to fluorescein isothiocyanate (FITC) and analyzed by flow cytometry

Peripheral blood mononuclear cells contain lymphocytes (B cells and T cells), monocytes, and fibrocytes precursors. Using immunomagnetic cell sorting, we found that the purity of fibrocytes originating from cultured PBMCs was almost 90 % (Fig. 1).

**Fig. 2:**
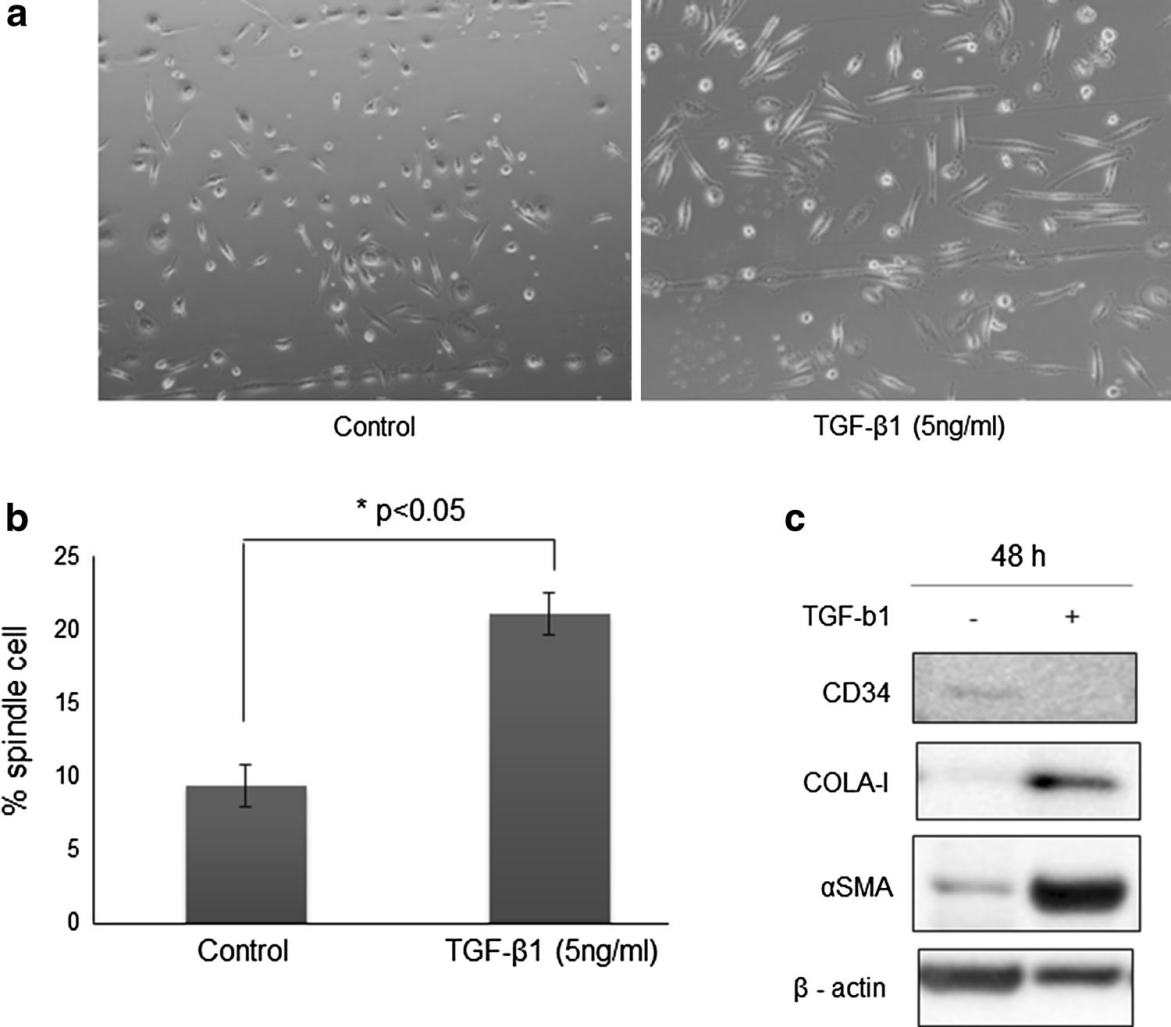
Morphology of fibrocytes isolated from cultures of peripheral blood mononuclear cells. **a** Cells cultured for 8 days in control medium (*left*). Cells cultured for 6 days in control medium and then for 2 days in control medium containing 5 ng/mL recombinant TGF-β1 (*right*). **b** Effects of TGF-β1 on fibrocyte differentiation in vitro. **c** Western blot analysis of the expression of CD34, collagen type I, and αSMA in these cultured cells. Each experiment was repeated three times, with the data expressed as means and standard deviations

Adherent cells from PBMCs preparations gradually elongated, showing a spindle-shaped morphology with an oval nucleus. The addition of TGF-β1 (5 ng/mL) to PBMC cultures on day 7 promoted fibrocytes differentiation, as shown by the increased percentage of cultured cells that were spindle shaped (Fig. 2a, b). In addition, western blotting analysis showed an increase in collagen type I and α-SMA expression, along with a decrease in CD34 expression (Fig. 2c). These results suggested that TGF-β1 promoted the differentiation of PBMCs into fibrocytes and myofibroblasts.

### Effect of co-culturing with gastric cancer cells and fibrocytes

**Fig. 3:**
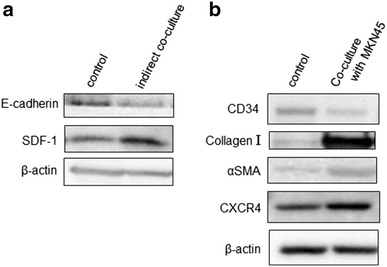
Co-culturing effects with MKN45 and fibrocytes. **a** Western blot analysis for E-cadherin and SDF-1 in MKN45 cells co-cultured with fibrocytes. **b** Western blot analysis for CD34, Collagen type I, αSMA, and CXCR4 in fibrocytes co-cultured with MKN45

Western blot analysis showed that indirect co-culturing of MKN45 cells with fibrocytes increased expression of SDF-1 and decreased expression of E-cadherin (Fig. 3a). In contrast, fibrocytes directly co-cultured with MKN45 cells and separated by immunomagnetic cell sorting showed increased expression of collagen type I, αSMA, and CXCR4, and decreased expression of CD 34 (Fig. 3b).

### Human gastric cancer cells co-cultured with fibrocytes significantly formed large mouse xenograft tumors

**Fig. 4:**
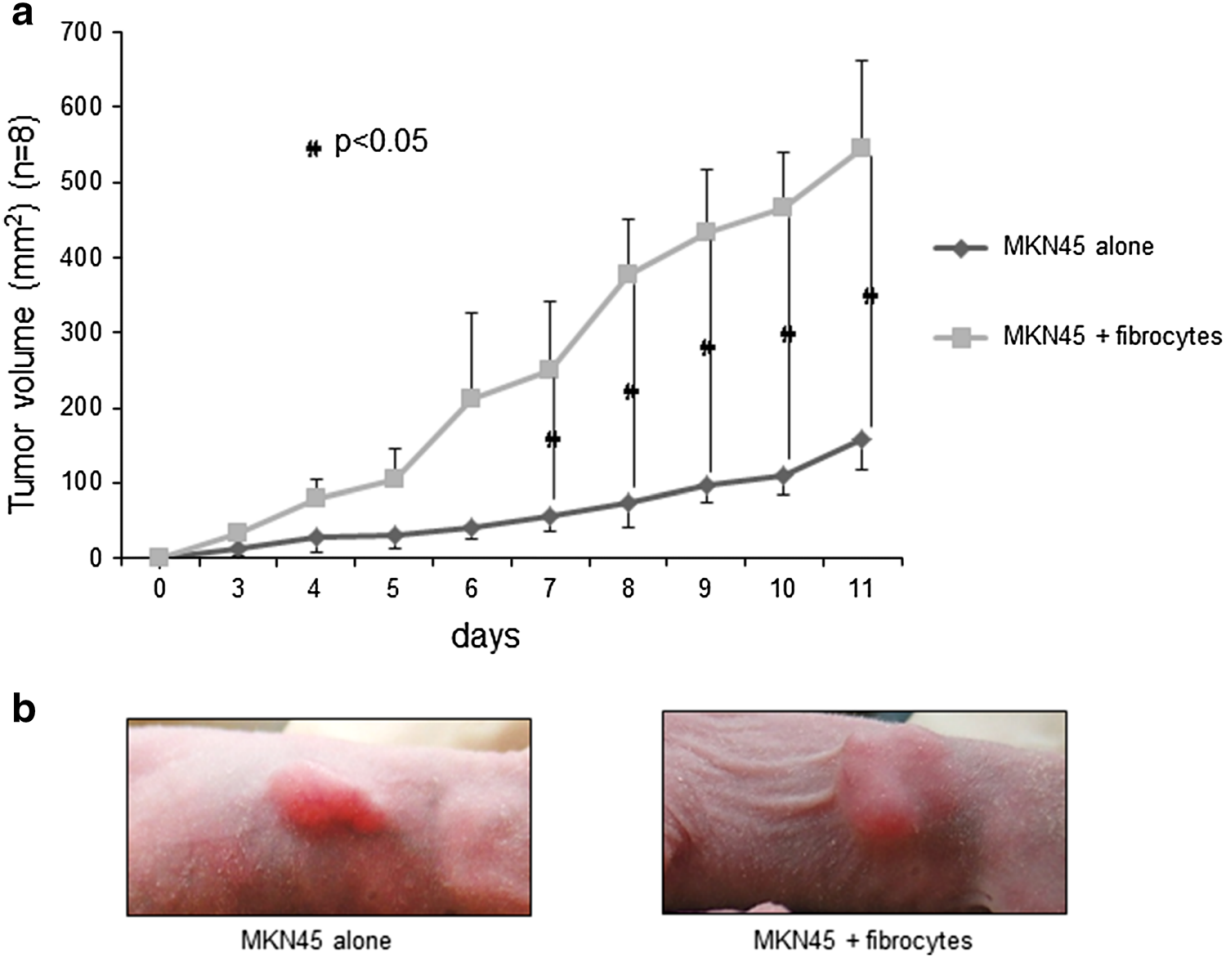
Growth of mouse xenograft models inoculated with MKN45 alone and MKN45 plus fibrocytes. Tumor volumes were calculated as the product of the length, width, and height of each tumor. Results are expressed as means and standard deviations (*n* = 8). **b** Representative images showing the macroscopic appearance of the tumors on day 11

**Fig. 5:**
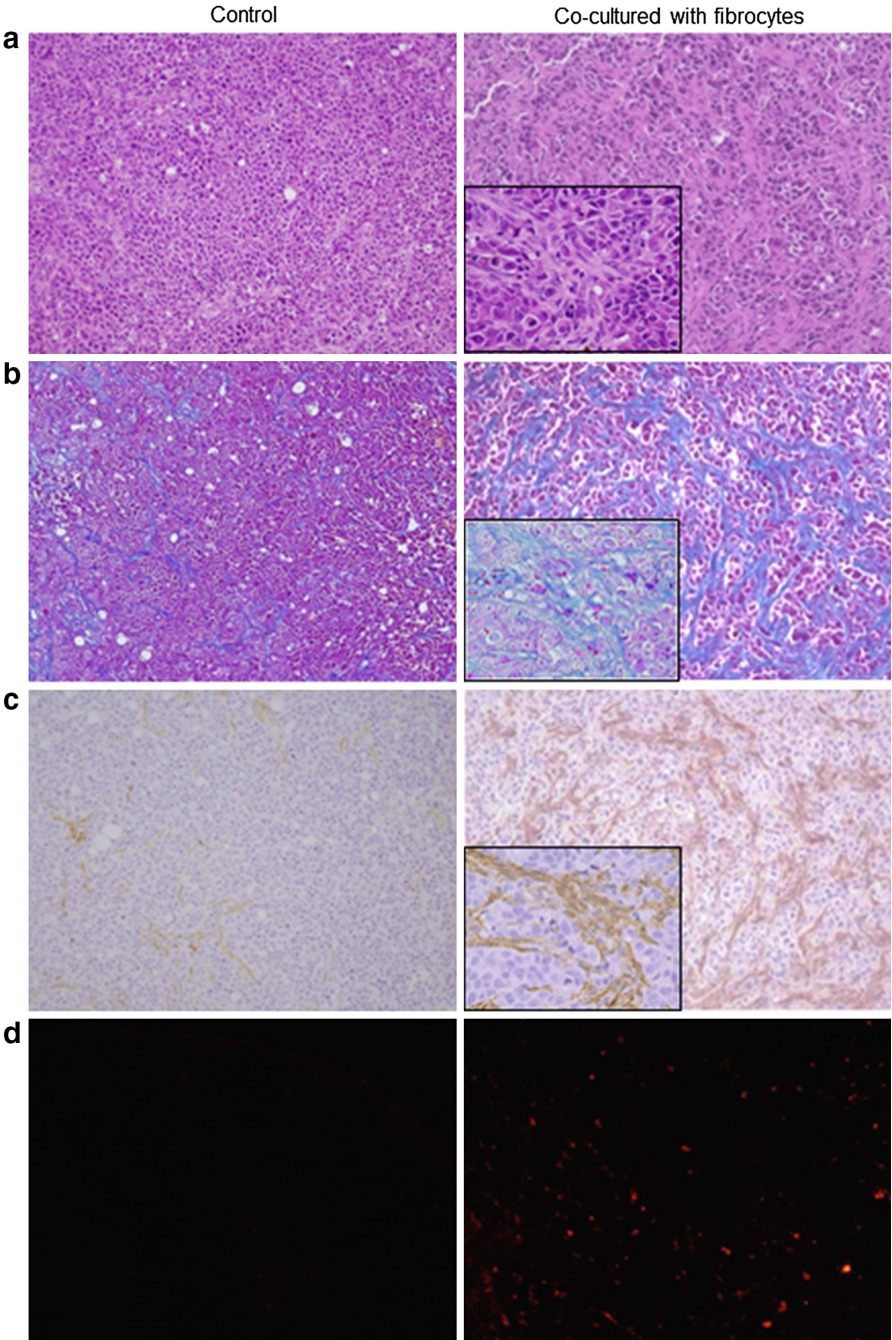
Microscopic views of mouse xenograft tumors (×200, and each magnification of enlarged view was ×400). **a** Histological appearance, as shown by H&E staining. **b** Fibrotic tissue, as determined by Mallory-Azan staining. **c** Immunohistochemical assays of αSMA expression. **d** Fluorescence microscopy results, showing the implantation of fibrocytes (*red*)

**Fig. 6:**
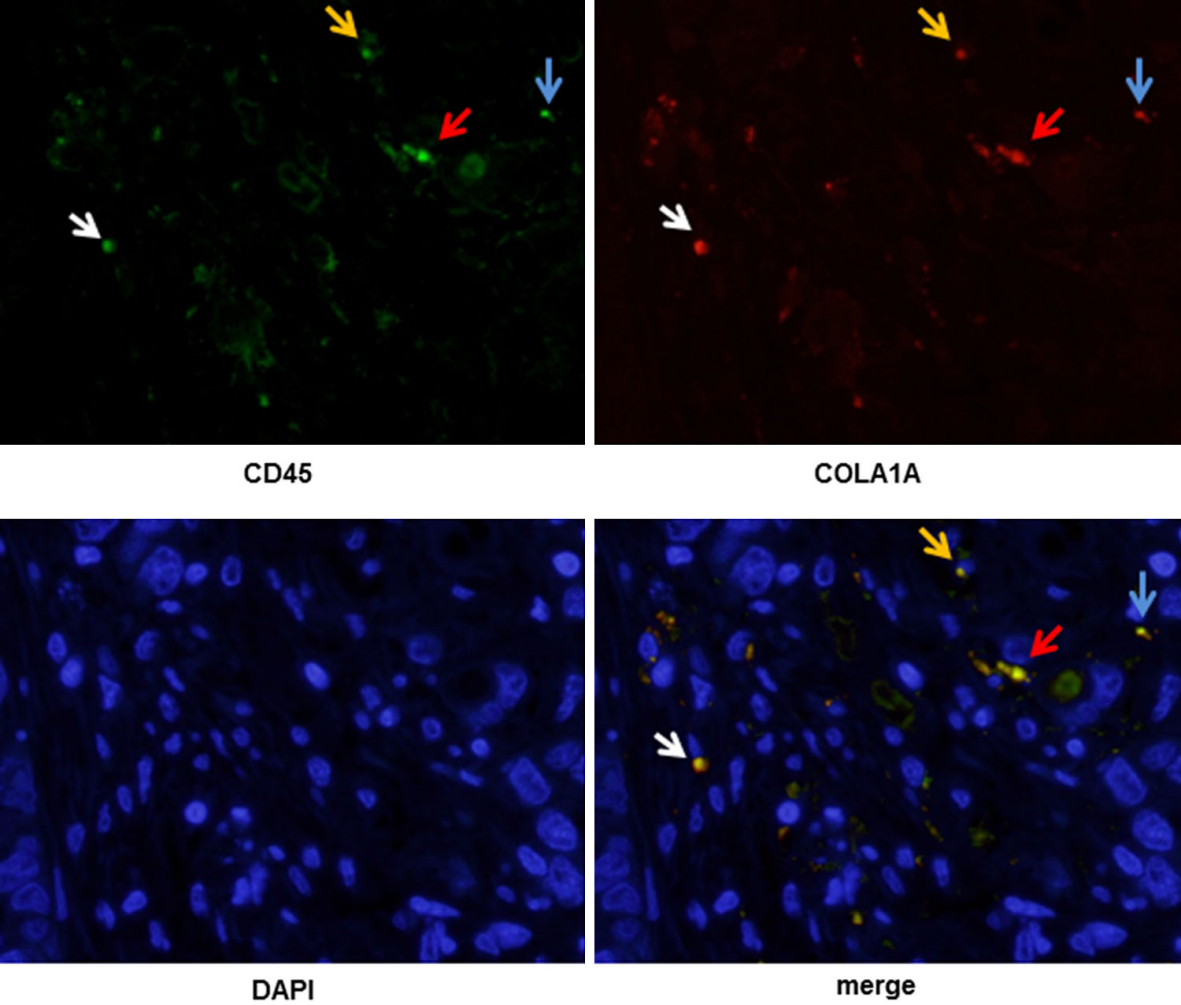
Dual immunohistochemical staining of mouse xenograft tumors with specific antibodies against CD45 and collagen type I, as shown by immunofluorescent microscopy (×400). *Each* *colored arrow* indicates a cell that is both mouse specific CD45 antigen positive and collagen type I positive. *Upper left* Immunostaining with rat anti-mouse CD45 antibodies (*green*). *Upper right* Immunostaining with rabbit anti-mouse collagen type I antibodies (*red*). *Lower left* Nuclear staining (*blue*). *Lower right* Merged images

The size of tumors from inoculated MKN45 cells plus fibrocytes was larger than that of MKN45 cells alone (Fig. 4), with the difference being statistically significant 7 days after inoculation (*p* < 0.05). The former tumors also had larger areas of fibrosis (Fig. 5a, b) and enhanced α-SMA expression (Fig. 5c). Moreover, a higher percentage of exogenous fibrocytes in co- inoculated tumors were positive for α-SMA expression (Fig. 5d). To determine whether endogenous host fibrocytes migrated into these tumors, the tumors were stained with antibodies to mouse COLA1A and CD45, antibodies that did not cross-react with human proteins. The co-inoculated tumors contained cells positive for both antigens, strongly indicating that mouse fibrocytes also participated in tumor formation (Fig. 6a–d).

## Discussion

We have shown that bone-marrow-derived circulating fibrocytes differentiated into myofibroblasts and contributed to tumor proliferation and fibrosis in a mouse xenograft model of gastric cancer. We also found that these tumors contained cells with the phenotypic characteristics of mouse bone marrow.

Cancer stroma is closely associated with the promotion of tumor growth and acquisition of invasive capacity. CAFs in tumors and surrounding tissues were recently reported to play important roles in the formation of tumor microenvironments [2, 7], including in gastric cancer. The percentage of orthotopic fibroblasts co-cultured with gastric cancer cells that differentiated into myofibroblasts was significantly higher than the percentage of normal orthotopic fibroblasts [19]. In addition, myofibroblasts in the microenvironment may stimulate gastric cancer cell motility, including migration and invasion [20]. Although the origins of CAFs have not yet been fully defined, they are thought to arise from multiple sites, such as neighboring tissues and remote organs. In addition, several studies have shown that bone marrow-derived cells are involved in the formation of cancer stroma [9, 21].

Peripheral blood circulating fibrocytes migrate to inflamed tissue sites in response to chemokines released by inflammatory cells [10], where they differentiate into fibroblasts and myofibroblasts and contribute to tissue inflammation and fibrosis formation [14, 22]. We found that co-culturing of fibrocytes with gastric cancer cells induced a fibroblast-like morphology in the former, and up-regulated their expression of αSMA and type I collagen, findings similar to those observed when PBMCs were cultured in the presence of TGF-β. Cancer cell-derived TGF-β has been reported to modulate myofibroblast differentiation in colon and breast cancer [23, 24], suggesting that cancer-cell-derived TGF-β may also induce differentiation of fibrocytes into myofibroblasts.

MKN45 cells were derived from hepatic metastatic tumor with a microscopic phenotype that was a solid type of poorly differentiated adenocarcinoma (por1). In this study, even MKN45 could acquire the ability of transforming fibrocytes. Most of gastric cancer cell lines were derived from various tumors that consist of cancer cells and stroma. Except for latent ability of stromal induction, MKN45 cells were considered to be appropriate in this study.

Subcutaneous co-implantation of MKN45 cells and fibrocytes formed significantly larger tumors and significantly greater stromal proliferation than implantation of MKN45 cells alone. The stroma in the co-implanted tumors contained collagen fibers and αSMA-expressing myofibroblasts, as well as a higher proportion of PKH26-positive cells. These findings indicated that bone marrow-derived fibrocytes may have contributed to the proliferation of xenograft tumors and differentiated toward myofibroblasts.

In addition, by using a subcutaneous model, it was possible to distinguish the migrated cells from orthotopic fibroblasts by mouse-specific antibodies. Immunohistochemical staining with antibodies against mouse CD45 and collagen type I showed a number of dual positive cells in co-implanted tumors. This result indicates that cancer stroma consisted not only of exogenous bone marrow-derived cells, but also of endogenous bone marrow-derived cells. In response to the chemokine SDF-1 released by inflammatory cells, fibrocytes expressing its receptor, CXCR4, migrated to inflamed sites to induce fibrosis [17, 25]. We confirmed that CXCR4 was expressed on fibrocytes and that SDF-1 was secreted by MKN45 cells in vitro. The presence of the SDF-1/CXCR4 system on these cells suggests that endogenous bone marrow-derived cells may also migrate to tumor sites and contribute to the formation of tumor stromal tissue in vivo.

Various cells other than CAFs contribute to the tumor microenvironment. For example, we have shown that activated peritoneal mesothelial cells co-cultured with MKN45 cells may promote tumor growth and fibrosis [26].

In conclusion, bone marrow-derived cells might migrate in a microenvironment of gastric cancer and differentiate into myofibroblasts. These myofibroblasts subsequently contribute to tumor formation and fibrosis as CAFs.
